# Latent class growth analysis of dynamic PaCo_2_ patterns and clinical outcomes in acute brain injury

**DOI:** 10.1038/s41598-025-04793-9

**Published:** 2025-05-30

**Authors:** Jian Liu, Bin Peng, Biao Huang, Fang Xu, Renjie Luo

**Affiliations:** 1 Department of Critical Care Medicine, The First Affiliated Hospital of Chongqing Medical University, Chongqing, P.R. China; 2 Department of Critical Care Medicine, Youyang Hospital, A Branch of The First Affiliated Hospital of Chongqing Medical University, Chongqing, P.R. China; 3 Department of Critical Care Medicine, Yunyang County People’s Hospital, Chongqing, PR China; 4 Department of Critical Care Medicine, The Affiliated Dazu’s Hopsital of Chongqing Medical University, Chongqing, P.R. China

**Keywords:** Acute brain injury, Arterial carbon dioxide partial pressure, Latent class growth analysis, Critical care, Neuromonitoring, Diseases of the nervous system, Neuro-vascular interactions

## Abstract

To analyze dynamic patterns of arterial carbon dioxide partial pressure (PaCO₂) using latent class growth analysis in acute brain injury patients and investigate their associations with 28-day ICU mortality and 60-day in-hospital mortality. This retrospective study utilized the Medical Information Mart for Intensive Care IV (MIMIC-IV) database. We applied latent class growth analysis to analyze PaCO₂ dynamic patterns during the first 72 h after ICU admission in adult patients with acute brain injury. Associations between trajectories and outcomes were evaluated using Cox proportional hazards models. Among the included acute brain injury patients (n = 1,145), three distinct PaCO₂ trajectories were identified: persistent hypocapnia pattern (23.7%), normal-mild regulation pattern (65.9%), and hypercapnia improvement pattern (10.5%). Cox proportional hazards regression analysis revealed that, compared with the normal-mild regulation pattern, the persistent hypocapnia pattern was significantly associated with higher risk of 28-day ICU mortality (HR = 1.28, 95% CI: 1.02–1.60) and 60-day in-hospital mortality (HR = 1.28, 95% CI: 1.03–1.59) after adjusting for confounding factors. The hypercapnia improvement pattern demonstrated a potentially protective association. This study identified distinct patterns of PaCO₂ dynamic changes in acute brain injury patients. Our analysis demonstrated that persistent hypocapnia was associated with higher risk of 28-day ICU and 60-day in-hospital mortality, while the hypercapnia improvement pattern showed potentially protective association. These results suggest the complexity of PaCO₂ management and the need for more individualized respiratory management strategies.

**Supplementary Information:**

The online version contains supplementary material available at 10.1038/s41598-025-04793-9.

## Introduction

Arterial carbon dioxide partial pressure (PaCO₂) is a crucial parameter for regulating cerebral hemodynamics, and its abnormal fluctuations can profoundly impact tissue perfusion and metabolism in patients with acute brain injury (ABI)^1,2^. Research has demonstrated that for each mmHg change in PaCO₂, the brain blood volume changes by 0.7 ml^3^. In severe traumatic brain injury, a mere 0.5 ml change in brain blood volume can result in a 1 mmHg change in intracranial pressure^4^. Clinicians commonly manipulate PaCO₂ levels by adjusting mechanical ventilation parameters to optimize intracranial pressure management. However, this intervention is a double-edged sword: while hyperventilation may temporarily reduce elevated intracranial pressure, it can lead to cerebral vasoconstriction and secondary ischemic injury. Conversely, sustained hypercapnia may exacerbate intracranial hypertension and cerebral edema through vasodilation, creating a vicious cycle.

Despite the clinical importance of PaCO₂ management, the optimal management strategy remains controversial. The fourth edition of the Brain Trauma guidelines acknowledges the lack of high-quality evidence supporting PaCO₂ management strategies in traumatic brain injury (TBI) patients^5^. The guidelines recommend against prophylactic hyperventilation and maintaining PaCO₂ ≤ 25 mmHg for prolonged periods but do not provide definitive conclusions regarding the ideal PaCO₂ target range. The 2019 Seattle International Severe Traumatic Brain Injury Consensus Conference recommends that mild hyperventilation (PaCO₂ 32–35 mmHg) should be considered as a second-line intervention only when first-line measures (sedation, osmotic therapy) prove ineffective and explicitly opposes routine reduction of PaCO₂ < 30 mmHg^6^. The 2020 expert consensus on ventilation management in acute brain injury emphasizes avoiding hypercapnia and recommends maintaining PaCO₂ within the physiological range (35–45 mmHg) as a basic management goal^7^. While these guidelines provide a clinical decision-making framework, they have yet to address the challenge of individualizing PaCO₂ management.

Existing research further highlights the complexity of PaCO₂ management. Robba et al. revealed a “U-shaped” relationship between PaCO₂ and in-hospital mortality, with both extremely low and high values associated with increased mortality^8^. However, the CENTER-TBI study revealed no significant association between even profound hypocapnia (PaCO₂ < 30 mmHg) and 6-month mortality^9^. These conflicting findings may stem from methodological differences, but more importantly, these studies focused primarily on the relationship between static PaCO₂ values and outcomes without adequately considering the dynamic characteristics of PaCO₂ abnormalities, such as duration, amplitude of fluctuation, and rate of change, which may have more profound physiological effects on brain tissue.

Latent class growth analysis (LCGA) can reveal heterogeneity that traditional static analyses fail to capture by identifying unique temporal patterns, thereby enabling more precise associations between dynamic patterns and clinical outcomes^10^. Proust-Lima et al. highlighted that this method offers unique advantages in studying disease progression and treatment responses^11^. We aimed to analyze PaCO₂ dynamic change patterns in ABI patients using the Medical Information Mart for Intensive Care IV (MIMIC-IV) database via LCGA and explore the associations between those trajectories and intensive care unit (ICU) outcomes (including 28-day ICU mortality and 60-day hospital mortality).

## Methods

### Data source and study population

This study was conducted using the publicly available MIMIC-IV database version 3.1, which contains deidentified health records from the Beth Israel Deaconess Medical Center. As the database is fully anonymized and publicly accessible for research purposes, the requirement for informed consent was waived. The authors completed the required training and obtained authorization to access the database (certification number: 11522514)^12^.

The inclusion criteria were as follows:

(1) Age ≥ 18 years;

(2) ABI identified via the ICD-10/ICD-9-CM codes for TBI (S06/800–801, 851–854), subarachnoid hemorrhage (I60/430), intracerebral hemorrhage (I61, I62/431, 432), and ischemic stroke (I63/433, 434); and.

(3) The first available PaCO_2_ measurement from arterial blood gas analysis was collected for each of three time periods: from 6 h preadmission to 24 h post-ICU admission (Day 1), 24–48 h after ICU admission (Day 2), and 48–72 h after ICU admission (Day 3).

Patients were excluded if they had chronic pulmonary disease or if PaCO₂ measurements were missing for any of the three time periods specified in criterion 3 above. Missing data < 30% were imputed via multivariate singular value decomposition imputation.

### Data collection

The data extracted from the database include the following: PaCO₂ measurements obtained through daily arterial blood gas analyses during the initial three days of ICU admission; demographic characteristics, including age, sex, height, weight and race; laboratory parameters, including hemoglobin concentration, serum albumin levels, and creatinine values; disease severity scoring systems, including the Acute Physiology Score III (APSIII), Sequential Organ Failure Assessment (SOFA), Glasgow Coma Scale (GCS), and Charlson Comorbidity Index; and comorbidities (diabetes, chronic kidney disease, congestive heart failure, and severe liver disease). We also extracted mechanical ventilation requirements, duration of mechanical ventilation, intracranial pressure monitoring, ICU length of stay, hospital length of stay, 28-day ICU mortality, 60-day in-hospital mortality, ICU mortality, and overall hospital mortality.

### Objectives

The primary objective was to identify distinct dynamic patterns of PaCO₂ changes within the first 3 days of ICU admission using latent trajectory analysis and to describe the clinical characteristics of each trajectory pattern. The secondary objective was to evaluate the associations of distinct PaCO₂ trajectories with 28-day ICU mortality and 60-day in-hospital mortality.

### Statistical analysis

For granular visualization of PaCO₂ transitions, daily measurements were classified into five categories: normocapnia (35–45 mmHg), mild hypocapnia (32–35 mmHg), severe hypocapnia (26–<32 mmHg), forced hypocapnia (< 26 mmHg), and hypercapnia (> 45 mmHg)^8^. These standardized thresholds enabled the construction of Sankey diagrams to track individual patient transitions between PaCO₂ categories across consecutive ICU days.

### Latent class growth analysis

LCGA was performed to identify distinct trajectories of PaCO_2_ dynamics during the initial 72 h of ICU admission. The model included fixed effects (linear and quadratic terms for time) to capture the average trajectory pattern within each class, assuming homogeneous growth parameters within classes without random effects. The optimal number of trajectory classes was selected through Bayesian information criterion (BIC) minimization, posterior class probabilities exceeding 0.70, minimum class size thresholds (≥ 2% of the cohort), and clinical interpretability.

### Baseline characterization

Continuous variables are summarized as the means ± standard deviations or medians (interquartile ranges) on the basis of distributional normality, whereas categorical variables are expressed as frequencies (percentages). Between-trajectory analyses employed parametric (one-way ANOVA) or nonparametric (Kruskal-Wallis) approaches for continuous variables based on distribution normality. Categorical comparisons initially utilized Pearson’s χ² tests supplemented by Fisher’s exact methods where low expected cell frequencies (< 5) occurred. Post hoc analyses were stratified by data characteristics: Tukey’s honestly significant difference (HSD) addressed parametric pairwise comparisons, Dunn tests with Bonferroni adjustment managed nonparametric contrasts, while categorical multiplicity control was achieved through α-level correction of contingency table analyses (χ²/Fisher’s tests with Bonferroni adjustment).

### Subgroup and sensitivity analyses

Given the probabilistic assignment of individuals to trajectory classes, sensitivity analyses were conducted to assess the robustness of trajectory classification. Subgroups were stratified by age (< 60 vs. ≥60 years), sex, race (white vs. nonwhite), Charlson Comorbidity Index (< 5 vs. ≥5), GCS score (< 12 vs. ≥12), comorbidities (presence/absence of diabetes, kidney disease, or congestive heart failure), and etiology of brain injury (traumatic vs. nontraumatic).

### Survival analysis

Survival differences across trajectory classes were assessed via Kaplan‒Meier curves with log-rank tests for both 28-day ICU mortality and 60-day in-hospital mortality. Time-to-event analyses employed multivariable Cox proportional hazards regression with hierarchical adjustment: Model 1 included demographic variables (age, sex, race); Model 2 incorporated the covariates from Model 1 plus illness severity indices and key laboratory parameters; Model 3 extended Model 2 by adding the comorbidities (diabetes, kidney disease, congestive heart failure), and mechanical ventilation requirement. The proportional hazards assumption was verified using Schoenfeld residuals.

All analyses were performed using R software (version 4.4.1). A two-sided *P* < 0.05 was considered to indicate statistical significance.

## Results

**Fig. 1:**
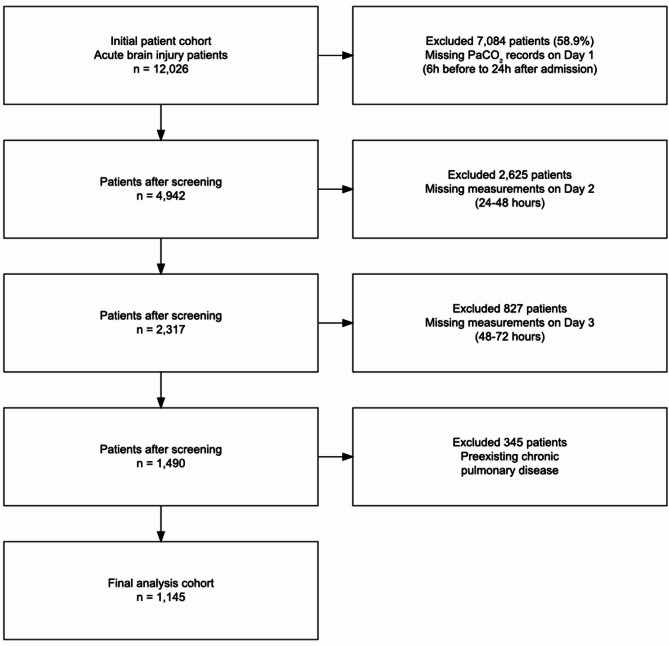
Patient selection flow diagram.

We extracted data on 12,026 patients with ABI from the MIMIC-IV v3.1 database. We sequentially applied the exclusion criteria: 7,084 patients (58.9%) were excluded because of missing PaCO₂ records on Day 1 (6 h before to 24 h after admission), followed by the exclusion of 2,625 and 827 patients because of missing measurements on Day 2 (24–48 h) and Day 3 (48–72 h), respectively. An additional 345 patients were excluded because of preexisting chronic pulmonary disease. The final cohort included 1,145 patients for analysis (Fig. 1). The mean age of the patients was 61.7 ± 17.6 years, with 696 males (60.8%) and 594 Whites (51.9%). There were 316 patients (27.6%) with TBI. The majority of patients (1,081 patients, 94.4%) received mechanical ventilation. Missing data were present for the following variables: weight (9.43%), height (97.73%), hemoglobin (0.26%), albumin (28%), creatinine (0.26%), GCS (0.79%), SOFA score (0.61%), and intracranial pressure monitoring (79.83%). Owing to the high percentage of missing height data required for BMI calculation, BMI was not included in the analysis.

### PaCO₂ trajectory patterns in ABI patients

**Fig. 2:**
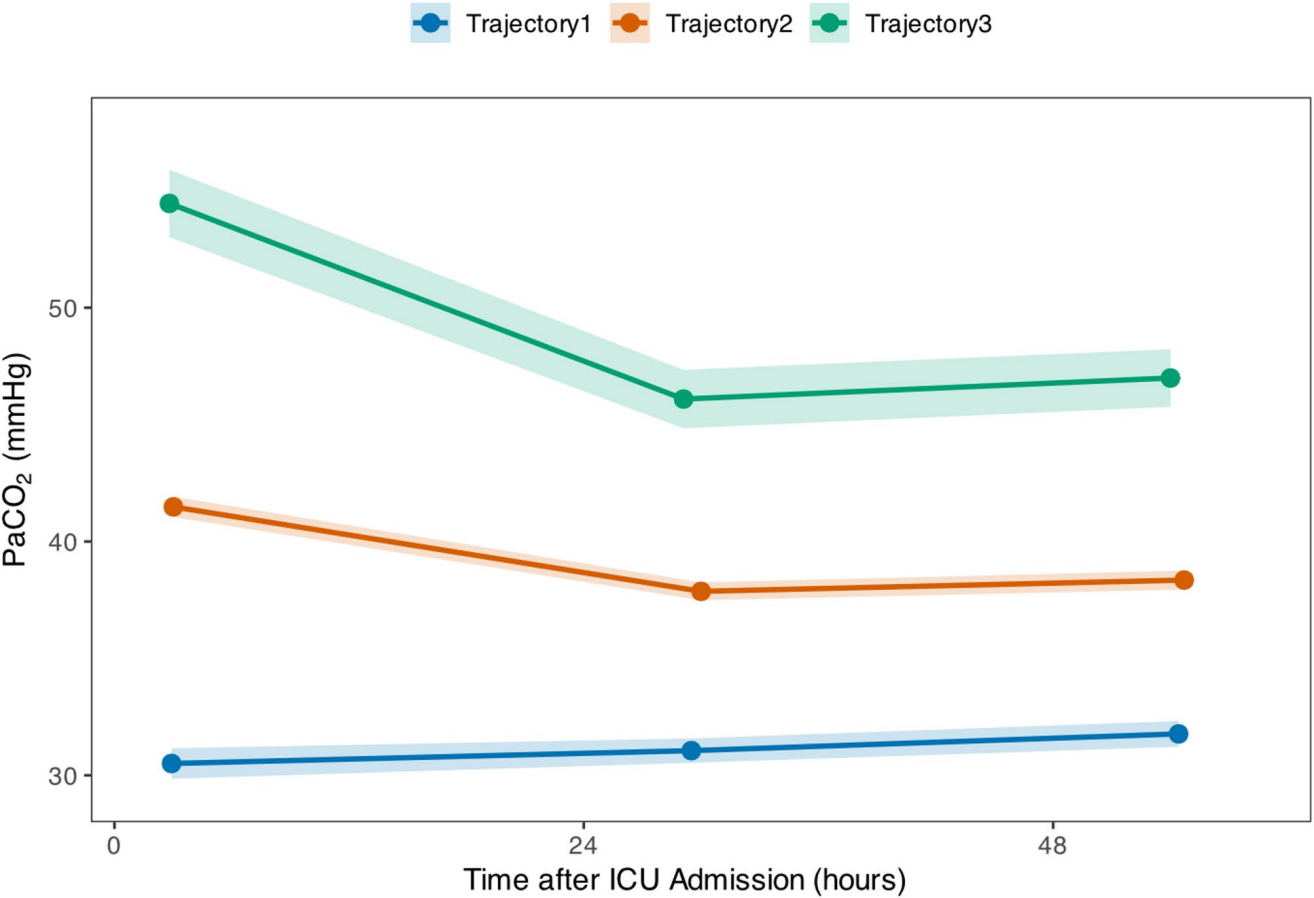
Three distinct PaCO₂ trajectories identified in acute brain injury patients during the first 3 day after ICU admission. Lines represent mean values with shaded areas indicating 95% confidence intervals (mean ± 1.96 × SD). Trajectory 1 (*n* = 271, 23.7%): Persistent hypocapnia pattern; Trajectory 2 (*n* = 754, 65.9%): Normal-mild regulation pattern; Trajectory 3 (*n* = 120, 10.5%): Hypercapnia improvement pattern.

Latent class growth analysis tested 2–7 class models. Although the 5-class model showed the lowest BIC value, considering classification quality, posterior group probabilities, and clinical interpretability, we selected the 3-class PaCO₂ trajectory model as the optimal solution (Table S1). The three distinct PaCO₂ trajectories identified in ABI patients are shown in Fig. 2.

Trajectory 1 - The persistent hypocapnia pattern (271, 23.7%) : Patients maintained consistently low PaCO₂ levels, slightly increasing from 30.5 ± 5.5 mmHg on Day 1 to 31.8 ± 4.6 mmHg on Day 3. Daily PaCO₂ ranges remained within a narrow interval, with means stabilizing between 30 and 32 mmHg throughout the observation period.

Trajectory 2 - The normal-mild regulation pattern (754, 65.9%): PaCO₂ decreased from 41.5 ± 6.1 mmHg on Day 1 to 37.9 ± 5.3 mmHg on Day 2, then maintained at 38.4 ± 5.7 mmHg on Day 3. This pattern, representing the majority of the study population, demonstrated a transition from slightly elevated levels to normal range with subsequent stabilization.

Trajectory 3 - The hypercapnia improvement pattern (120, 10.5%): PaCO₂ significantly decreased from marked elevation (54.5 ± 8.1 mmHg) on Day 1 to 46.1 ± 7.0 mmHg on Day 2, maintaining similar levels on Day 3 (47.0 ± 6.9 mmHg). This indicated partial improvement of initial hypercapnia, though most patients remained at mild hypercapnia levels.

### Sankey diagram visualization of paco₂ level changes across three trajectories

Figures S1-S3 show Sankey diagrams illustrating the daily transitions between PaCO₂ categories across the three identified trajectories.

Trajectory 1: Day 1 was characterized predominantly by hypocapnia, with 54% (146/271) of patients exhibiting severe or forced hypocapnia, while only 17% (47/271) showed normocapnia. On Day 2, there was further transition toward hypocapnia, with 79% (215/271) demonstrating severe or moderate hypocapnia. Day 3 patterns remained largely stable, though normocapnia slightly increased to 21% (58/271). The primary transitions reflected persistent hypocapnia with minor recovery to normocapnia.

Trajectory 2: Day 1 was dominated by normocapnia (60%, 450/754) with some hypercapnia (24%, 182/754). By Day 2, hypercapnia patients decreased markedly to 7.4% (56/754), while hypocapnia increased to 31% (237/754). Day 3 showed relative stability, with hypercapnia slightly increasing to 10.5% (79/754) and normocapnia remaining predominant (57%, 432/754). The most notable transition was from hypercapnia to normal and mild hypocapnia states.

Trajectory 3: Day 1 was characterized by predominant hypercapnia (86.7%, 104/120). On Day 2, hypercapnia decreased to 53% (64/120) with normocapnia increasing to 41% (49/120). Day 3 showed relative stability, with hypercapnia slightly rebounding to 56% (67/120) and normocapnia maintaining at 40% (48/120). The primary transition pattern demonstrated partial improvement from marked hypercapnia to normal or mild hypercapnic states.

### Comparison of baseline characteristics

Baseline characteristics across trajectories are summarized in Table 1. Significant differences were observed in demographic characteristics. Trajectory 1 patients were older (64.44 ± 16.42 years vs. trajectory 2: 61.04 ± 17.99 years vs. trajectory 3: 59.61 ± 16.99 years; *P* = 0.009) and had a lower proportion of males (53.1% vs. Trajectory 2: 62.7% vs. Trajectory 3: 65.8%; *P* = 0.010), while racial composition showed no significant differences (*P* = 0.401).

**Table 1:** Baseline characteristics of study participants by trajectory.

Characteristics	Overall (*n* = 1145)	Trajectory 1 (*n* = 271)	Trajectory 2 (*n* = 754)	Trajectory 3 (*n* = 120)	*P* value
Age (years)	61.70 (17.59)	64.44 (16.42)	61.04 (17.99)	59.61 (16.99)	0.009
Male, n (%)	696 (60.8)	144 (53.1)	473 (62.7)	79 (65.8)	0.010
Race, n (%)					0.401
White	594 (51.9)	133 (49.1)	402 (53.3)	59 (49.2)	
Non-White	551 (48.1)	138 (50.9)	352 (46.7)	61 (50.8)	
Hemoglobin (g/dL)	11.04 (2.37)	11.04 (2.45)	11.00 (2.38)	11.32 (2.10)	0.399
Albumin (g/dL)	3.05 (0.57)	3.08 (0.62)	3.07 (0.54)	2.92 (0.61)	0.018
Creatinine (mg/dL)	1.00 (0.80, 1.40)	1.00 (0.70, 1.55)	1.00 (0.80, 1.40)	1.10 (0.80, 1.80)	0.167
Charlson Comorbidity Index	5 (3, 7)	5 (3, 7)	5 (2, 7)	4 (2, 6)	0.010
APSIII	48 (35, 64)	50 (36, 70)	47 (34, 61)	53 (41, 75)	< 0.001
SOFA	6 (4, 8)	6 (3, 8)	6 (3, 8)	6 (4, 9)	0.027
GCS	15 (11, 15)	15 (10, 15)	15 (12, 15)	15 (10, 15)	0.096
Diabetes, n (%)	349 (30.5)	76 (28.0)	233 (30.9)	40 (33.3)	0.526
Renal Disease, n (%)	194 (16.9)	53 (19.6)	125 (16.6)	16 (13.3)	0.287
Congestive Heart Failure, n (%)	253 (22.1)	52 (19.2)	165 (21.9)	36 (30.0)	0.058
Severe Liver Disease, n (%)	46 (4.0)	12 (4.4)	30 (4.0)	4 (3.3)	0.875
Mechanical ventilation, n (%)	1081 (94.4)	247 (91.1)	719 (95.4)	115 (95.8)	0.027
Duration of mechanical ventilation (hours)	147 (82, 252)	146 (81, 251)	153 (80, 270)	139 (83, 256)	0.969
TBI Status					0.001
TBI	316 (27.6)	47 (17.3)	224 (29.7)	45 (37.5)	
Non-TBI	829 (72.4)	224 (82.7)	230 (70.3)	75 (62.5)	
LOS ICU (days)	9.76 (5.87, 16.60)	9.13 (5.39, 16.55)	9.84 (5.92, 16.74)	10.57 (6.20, 16.49)	0.376
LOS Hospital (days)	17.59 (10.11, 28.58)	16.28 (8.34, 27.34)	17.91 (10.56, 28.18)	17.99 (12.41, 29.41)	0.083
ICU mortality, n (%)	288 (25.2)	83 (30.6)	178 (23.6)	27 (22.5)	0.057
Hospital mortality, n (%)	371 (32.4)	108 (39.9)	230 (30.5)	33 (27.5)	0.009

Data are presented as No. (%) unless otherwise indicated. Continuous variables are presented as mean (SD) or median (IQR).

For binary variables (diabetes, renal disease, congestive heart failure, mechanical ventilation), proportions represent presence of the condition.

Abbreviations: APSIII, Acute Physiology Score III; GCS, Glasgow Coma Scale; ICU, intensive care unit; IQR, interquartile range; LOS, length of stay; SD, standard deviation; SOFA, Sequential Organ Failure Assessment; TBI, traumatic brain injury.

The prevalence of most comorbidities, including diabetes (*P* = 0.526), kidney disease (*P* = 0.287), and severe liver disease (*P* = 0.875), was similar across trajectory patterns, while congestive heart failure showed a trend toward higher prevalence in trajectory 3 (30.0% vs. trajectory 1: 19.2% vs. trajectory 2: 21.9%; *P* = 0.058). The distribution of TBI patients differed significantly among trajectories (*P* = 0.001), with trajectory 3 having the highest proportion (37.5%), followed by trajectory 2 (29.7%), and trajectory 1 having the lowest (17.3%). The requirement for mechanical ventilation showed significant differences (*P* = 0.027), while its duration remained similar (*P* = 0.969).

Disease severity indicators showed significant variations among trajectories. Trajectory 3 patients presented with significantly higher APSIII scores [53 (41–75) vs. trajectory 1: 50 (36–70) and trajectory 2: 47 (34–61); *P* < 0.001] and demonstrated differences in SOFA scores [6 (4–9) vs. trajectory 1: 6 (3–8) and trajectory 2: 6 (3–8); *P* = 0.027]. Serum albumin levels (g/dL) showed significant differences among trajectories (*P* = 0.018), with trajectory 3 demonstrating significantly lower levels (2.92 ± 0.61) compared to both trajectory 1 (3.08 ± 0.62) and trajectory 2 (3.07 ± 0.54).

While ICU and hospital length of stay showed no significant differences among trajectories (*P* = 0.376 and *P* = 0.083, respectively), hospital mortality rates demonstrated significant variations (*P* = 0.009). Trajectory 1 showed the highest hospital mortality rate (39.9%), followed by trajectory 2 (30.5%), and trajectory 3 having the lowest (27.5%). Although ICU mortality rates did not reach statistical significance, they showed a similar trend with trajectory 1 having the highest rate (30.6% vs. trajectory 2: 23.6% vs. trajectory 3: 22.5%; *P* = 0.057).

### Survival outcome analysis

**Fig. 3:**
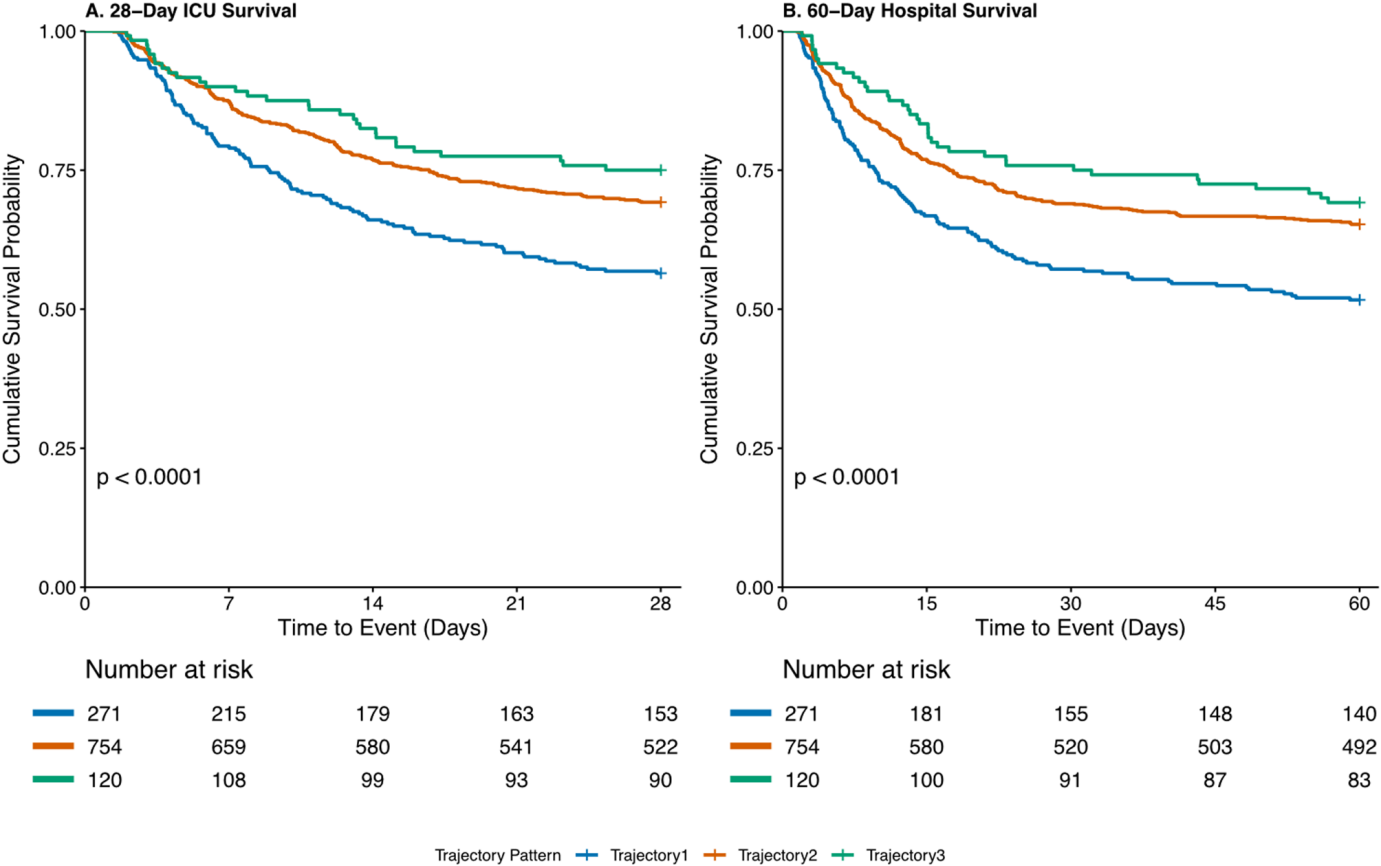
Kaplan − Meier Survival Estimates Stratified by PaCO₂ Trajectory Patterns. Kaplan-Meier analysis of ICU 28-day survival showed significant differences among the three trajectory patterns (Fig. 3A, log-rank χ² = 20.4, *p* < 0.0001). Similarly, hospital 60-day mortality demonstrated significant differences (Fig. 3B, log-rank χ² = 20.6, *p* < 0.0001).

Trajectory pattern analysis revealed significant survival differences among the three PaCO₂ trajectories. Kaplan-Meier analysis demonstrated significantly lower survival rates in trajectory 1 compared to trajectories 2 and 3 for ICU 28-day and hospital 60-day outcomes (both *P* < 0.0001) (Fig. 3).

Cox proportional hazards analysis was performed using trajectory 2 as the reference group for both ICU 28-day mortality and hospital 60-day mortality outcomes (Table 2).

**Table 2:** Univariate and multivariate Cox regression analysis of the three PaCO2 Trajectories.

Outcome	Group	Crude	Model I	Model II	Model III
ICU-28-day mortality	Trajectory 2	Ref	Ref	Ref	Ref
	Trajectory 1	1.56 (1.25−1.95, *p* < 0.001)	1.40 (1.12–1.76, *p* = 0.003)	1.29 (1.03–1.62, *p* = 0.025)	1.28 (1.02–1.60, *p* = 0.036)
	Trajectory 3	0.78 (0.53–1.14, *p* = 0.205)	0.79 (0.54–1.16, *p* = 0.236)	0.70 (0.47–1.02, *p* = 0.066)	0.69 (0.47–1.01, *p* = 0.059)
hosp-60-day mortality	Trajectory 2	Ref	Ref	Ref	Ref
	Trajectory 1	1.55 (1.26–1.91, *p* < 0.001)	1.38 (1.12–1.71, *p* = 0.003)	1.28 (1.03–1.59, *p* = 0.023)	1.28 (1.03–1.59, *p* = 0.025)
	Trajectory 3	0.84 (0.60–1.19, *p* = 0.321)	0.84 (0.60–1.19, *p* = 0.334)	0.74 (0.52–1.05, *p* = 0.097)	0.75 (0.53–1.06, *p* = 0.104)

Model I adjusted for age, gender, and race.

Model II adjusted all Model I covariates plus disease severity scores (APSIII, SOFA, GCS), and laboratory parameters (hemoglobin, albumin, creatinine).

Model III adjusted for all Model II covariates plus comorbidities (diabetes, renal disease, congestive heart failure) and clinical characteristics (mechanical ventilation).

For ICU 28-day mortality, trajectory 1 was significantly associated with increased mortality risk across all models. In the unadjusted analysis, the HR was 1.56 (95% CI: 1.25–1.95, *P* < 0.001). After adjusting for demographic factors (Model I), the HR decreased to 1.40 (95% CI: 1.12–1.76, *P* = 0.003). Additional adjustment for disease severity and laboratory indicators (Model II) yielded a HR of 1.29 (95% CI: 1.03–1.62, *P* = 0.025). The fully adjusted model (Model III) maintained statistical significance (HR = 1.28, 95% CI: 1.02–1.60, *P* = 0.036). Notably, trajectory 3 showed a potential protective association, approaching statistical significance in the fully adjusted model (HR = 0.69, 95% CI: 0.47–1.01, *P* = 0.059).

Similar patterns were observed for hospital 60-day mortality. Trajectory 1 was consistently and significantly associated with increased mortality risk, from the unadjusted analysis (HR = 1.55, 95% CI: 1.26–1.91, *P* < 0.001) through to the fully adjusted model (HR = 1.28, 95% CI: 1.03–1.59, *P* = 0.025). While trajectory 3 was associated with a trend toward lower risk compared to the reference group, particularly after adjusting for disease severity and laboratory indicators (Model II: HR = 0.74, 95% CI: 0.52–1.05, *P* = 0.097), this protective association did not reach statistical significance in the fully adjusted model (HR = 0.75, 95% CI: 0.53–1.06, *P* = 0.104).

### Subgroup analysis

To assess the robustness of our findings, stratified analyses were conducted across age (< 60 vs. ≥60 years), gender, race (white vs. nonwhite), Charlson comorbidity index (< 5 vs. ≥5), GCS score (< 12 vs. ≥12), diabetes, chronic kidney disease, congestive heart failure, and TBI status (Figure S4, Figure S5).

The results demonstrated that trajectory 1, compared to the reference group (trajectory 2), exhibited elevated mortality risk across most subgroup analyses. Two notable findings emerged from the stratified analyses: First, age-stratified analysis revealed significant interaction effects (ICU 28-day: *P* = 0.025; hospital 60-day: *P* = 0.03), with stronger associations between trajectory 1 and mortality risk observed in younger patients (< 60 years) (ICU 28-day: HR = 2.23, 95% CI: 1.46–3.39, *P* < 0.001; hospital 60-day: HR = 2.14, 95% CI: 1.44–3.20, *P* < 0.001). Second, stratification by TBI status demonstrated significant interaction effects (ICU 28-day: *P* = 0.038; hospital 60-day: *P* = 0.012), with stronger associations observed in TBI patients (ICU 28-day: HR = 2.71, 95% CI: 1.64–4.46, *P* < 0.001; hospital 60-day: HR = 2.80, 95% CI: 1.73–4.54, *P* < 0.001).

Stratified analyses of other clinical characteristics showed no significant effect modification, indicating the stability of associations between PaCO₂ trajectories and outcomes across subgroups. These findings not only supported the primary analysis but also identified younger patients and TBI patients as high-risk subgroups.

## Discussion

This study represents the application of LCGA to analyze dynamic PaCO₂ fluctuation patterns in ABI patients. Our analysis revealed three distinct trajectory clusters: the persistent hypocapnia pattern (23.7%), the normal-mild regulation (65.9%), and the hypercapnia improvement pattern (10.5%). Persistent hypocapnia pattern showed statistically significant associations with 28-day ICU mortality and 60-day in-hospital mortality, even after comprehensive adjustment for potential confounders, including demographic characteristics, disease severity indices, and comorbidities. These findings suggest that the temporal evolution of PaCO₂ may provide more nuanced insights into the complex relationship between carbon dioxide management strategies and clinical outcomes in ABI patients. This trajectory-based approach offers a novel perspective for understanding PaCO₂ dynamics in neurological critical care.

The three distinct trajectories identified in our study delineate divergent evolutionary patterns of PaCO₂ in acute brain injury patients. As illustrated by Sankey diagrams, trajectory 1 (23.7%) demonstrated persistent hypocapnia, with over half of the patients exhibiting severe or forced hypocapnia on Day 1, a pattern that persisted throughout the observation period. Trajectory 2 (65.9%) showed a transition from mild hypercapnia to normal values with subsequent stabilization. This evolution aligns with current ventilation management guidelines^5^. Trajectory 3 (10.5%) represented a progression from marked hypercapnia to partial improvement, though most patients maintained mild hypercapnic levels.

Notably, trajectory 1 was associated with significantly increased mortality risk, consistent with Roberts et al.‘s systematic review, which indicated that abnormal PaCO₂ levels following brain injury were associated with poor outcomes^13^.Our findings particularly emphasize that sustained hypocapnia may be a key factor contributing to increased mortality risk. This may be attributed to cerebral vasoconstriction associated with hypocapnia, which could lead to temporary reductions in cerebral blood flow and cerebral oxygenation^14,15^.

Furthermore, we observed that trajectory 3 demonstrated a potentially protective trend. This finding resonates with Curley et al.‘s research, which emphasized that optimal PaCO₂ strategies should be tailored based on underlying pathophysiological mechanisms^16^. Chesnut et al. also noted that brain injury treatment should be based on overall pathophysiological mechanisms rather than isolated parameters^17^. The gradual improvement from initial hypercapnia may reflect a more adaptive physiological regulation process, avoiding abrupt changes induced by aggressive ventilation. As reported by Spaite et al., in traumatic brain injury patients, preventing rapid PaCO₂ fluctuations may be more crucial for outcomes than strict adherence to specific target values^18^.

Our findings present an interesting contrast with two recent studies. A multicenter investigation by Robba et al. (*n* = 1476) revealed a U-shaped relationship between PaCO₂ and in-hospital mortality, where both extremely low (< 32 mmHg) and high (> 45 mmHg) values were associated with increased mortality risk^8^. However, our latent trajectory analysis revealed that when dynamic PaCO₂ patterns rather than static extreme values were considered, more nuanced and clinically meaningful associations emerged. Our trajectory modeling approach, particularly the identification of sustained hypocapnia, provides a dynamically sensitive indicator for prognostic assessment in ABI patients.

In contrast, the CENTER-TBI study (*n* = 1100) revealed no significant association between hypocapnia and 6-month mortality (OR = 1.06, 95% CI = 0.77–1.45; *P* = 0.716)^9^. This discrepancy may stem from methodological differences—point-to-point measurements versus temporal evolution pattern analysis. Particularly noteworthy is the CENTER-TBI study’s documentation of substantial intercenter variability in PaCO₂ management strategies, reflecting the current uncertainty in optimal carbon dioxide regulation protocols.

Our stratified analyses revealed important interaction effects, particularly the influence of age (< 60 years) and TBI status on the association between trajectory 1 and mortality risk. This suggests that certain patient subgroups may be more sensitive to PaCO₂ management strategies, further supporting the necessity of individualized approaches.

The 2022 European Society of Intensive Care Medicine consensus strongly recommends maintaining PaCO₂ between 35 and 45 mmHg in ABI patients without significant intracranial hypertension, although this recommendation is based on low-quality evidence^6^. Our trajectory analysis, revealing associations between sustained hypocapnia and poor outcomes, supports the recommendation against excessive ventilation. Smith et al. suggested that PaCO₂ management strategies should be differentiated among patients with increased intracranial pressure: hyperventilation therapy might be appropriate for those with vasodilation and vascular congestion, whereas it should be avoided in patients with intracranial hypertension due to other pathological mechanisms^19^. Such longitudinal modeling, through delineating the dynamic evolution patterns of PaCO₂ trajectories, provides a novel temporal dynamic perspective to this pathophysiological discourse.

However, these findings cannot establish causality. As Zhang et al. emphasized in their study on causal inference in longitudinal clinical data, establishing causal relationships in complex clinical settings presents significant challenges^20^. In our study, PaCO₂ trajectories may be simultaneously influenced by clinical interventions, underlying pathophysiological conditions, and patient baseline characteristics, while potentially mediating outcomes. Traditional regression adjustments cannot fully account for these complex temporal relationships. Therefore, our findings should be viewed as hypothesis-generating rather than definitive evidence supporting specific management strategies.

Our subgroup analyses demonstrated that the associations between PaCO₂ trajectory classification and clinical outcomes maintained directional consistency in their primary effects. Notably, although we excluded patients with chronic pulmonary diseases to reduce confounding, patient heterogeneity remains a crucial consideration in PaCO₂ management. Patients with different disease states may require differentiated PaCO₂ management strategies, which might explain why standardized PaCO₂ targets have not shown uniform effects in improving outcomes. Curley et al. emphasized that a “one-size-fits-all” PaCO₂ target may not be suitable for all patients, particularly when multiple pathophysiological mechanisms coexist^16^.

Hong et al. recently proposed a methodological framework for clinical phenotyping, demonstrating how routine clinical data can be utilized to identify disease heterogeneity and guide individualized treatment strategies^21^. This approach has important implications for PaCO₂ management in mechanically ventilated patients with ABI. By integrating physiological parameters, disease characteristics, and treatment responses, it may be possible to identify optimal PaCO₂ trajectories for specific patient subgroups.

### Limitations and future directions

This study has several limitations. Firstly, as a retrospective study, we encountered specific selection biases: including unbalanced PaCO₂ measurement frequency across different clinical scenarios that might affect trajectory construction, and systematic differences in patient baseline characteristics that might confound the associations between trajectories and outcomes due to clinical decisions on ventilation strategies. Furthermore, the absence of standardized neurological function assessments, particularly validated scales such as the Glasgow Outcome Scale-Extended (GOS-E) or modified Rankin scale (mRS), limits our ability to evaluate subtle neurological outcomes. Additionally, while PaCO₂ trajectories were thoroughly analyzed, we did not incorporate comprehensive multimodal neuromonitoring data, including intracranial pressure dynamics, brain tissue oxygenation, and cerebral blood flow measurements, which could provide crucial context for understanding the physiological implications of different PaCO₂ patterns.

We suggest two directions worthy of future investigation. First, prospective studies are needed to validate the reproducibility of these three PaCO₂ trajectories and explore their relationship with neurological recovery. As Helbok et al. noted, traditional outcome measures (such as mortality) may not fully reflect the benefits of neuroprotective interventions^22^. Second, the integration of high-frequency multimodal neuromonitoring data, including cerebral autoregulation status, brain tissue oxygenation, and metabolic parameters, could reveal critical physiological interactions that mediate the effects of PaCO₂ management^23^.

## Conclusions

This study innovatively applied LCGA to reveal heterogeneous patterns of dynamic changes in PaCO₂ in patients with ABI. Our findings showed that sustained hypocapnia was significantly associated with increased risk of ICU 28-day mortality and hospital 60-day mortality, while the hypercapnia improvement pattern showed a potentially protective trend. These findings emphasize the complexity of PaCO₂ management and the necessity of individualized treatment. The associations between PaCO₂ trajectories and outcomes were more pronounced in younger patients and the TBI subgroup, providing new clinical perspectives for optimizing respiratory management strategies in neurocritically ill patients.

## Electronic supplementary material

Below is the link to the electronic supplementary material.

Supplementary Material 1

## Data Availability

The data that support the findings of this study are available in the MIMIC-IV v3.1 database (https://physionet.org/content/mimiciv/3.1/). Due to patient privacy protection requirements, researchers need to complete necessary training and obtain authorization before accessing the database. Interested researchers can apply for access through the PhysioNet platform (https://physionet.org/). For specific inquiries about the data used in this study, please contact the corresponding author (F.X., xufang828@126.com).
