# Supplementary material for: Latent class growth analysis of dynamic PaCo2 patterns and clinical outcomes in acute brain injury
Source: Sci Rep. 2025 May 30;15:19100. doi: 10.1038/s41598-025-04793-9 (PMC12125163; doi:10.1038/s41598-025-04793-9)
Supplement: Supplementary file 1 — Supplementary Material 1 [file 41598_2025_4793_MOESM1_ESM.docx]

Table S1. **Model Fit Comparison of Latent Trajectory Models for PaCO₂ Dynamics in Acute Brain Injury Patients**

| **Number of trajectories** | **BIC** | **Group Size (%)** | **AvePP of each trajectory (%)** | **Proportion with Posterior Probability > 0.7** |
| --- | --- | --- | --- | --- |
| 1 | 23794.14 | 100.0 | 100.0 | 100.0 |
| 2 | 23440.90 | 70.8, 29.2 | 90.2, 83.6 | 88.4, 74.6 |
| 3 | 23368.52 | 23.6, 65.9, 10.5 | 80.0, 82.4, 82.9 | 73.8, 80.2, 75.0 |
| 4 | 23276.49 | 21.0, 63.5, 7.5, 8.0 | 75.6, 83.3, 80.5, 82.8 | 67.5, 81.7, 68.6, 71.7 |
| 5 | 23247.62 | 15.4, 13.3, 59.4, 9.7, 2.2 | 78.3, 74.8, 80.6, 79.7, 86.8 | 64.2, 60.8, 78.2, 68.5, 84.0 |
| 6 | 23271.53 | 16.6, 12.8, 58.4, 0.6, 9.5, 2.1 | 78.7, 74.2, 80.6, 77.5, 80.7, 87.8 | 64.2, 60.9, 79.1, 85.7, 70.6, 87.5 |
| 7 | 23281.78 | 5.1, 9.7, 45.5, 30.2, 0.8, 5.6, 3.1 | 78.9, 71.9, 76.4, 66.9, 75.1, 78.5, 82.3 | 31.3, 0, 48.4, 33.7, 57.1, 74.3, 51.1 |

Abbreviation: BIC, Bayesian Information Criterion; AvePP, Average posterior probability.

**Figure S1.** Sankey Diagram Visualization of Daily PaCO₂ Level Changes in Trajectory 1.

**Figure S2.** Sankey Diagram Visualization of Daily PaCO₂ Level Changes in Trajectory 2.

**Figure S3.** Sankey Diagram Visualization of Daily PaCO₂ Level Changes in Trajectory 3.

**Figure S4.** Subgroup analysis of 28-day ICU mortality.

**Figure S5.** Subgroup analysis of 60-day hospital mortality.
